# Supplementary material for: SyNPL: Synthetic Notch pluripotent cell lines to monitor and manipulate cell interactions in vitro and in vivo
Source: Development. 2022 Jun 23;149(12):dev200226. doi: 10.1242/dev.200226 (PMC9270970; doi:10.1242/dev.200226)
Supplement: Reviewer comments [file develop-149-200226_review_history.pdf]

SyNPL: Synthetic Notch pluripotent cell lines to monitor and manipulate cell interactions *in vitro* and *in vivo*

Mattias Malaguti, Rosa Portero Migueles, Jennifer Annoh, Daina Sadurska, Guillaume Blin and Sally Lowell

DOI: 10.1242/dev.200226

Editor: James Briscoe

Review timeline

Original submission:	24 September 2021
Editorial decision:	4 November 2021
First revision received:	7 April 2022
Editorial decision:	3 May 2022
Second revision received:	11 May 2022
Accepted:	13 May 2022

Original submission

First decision letter

MS ID#: DEVELOP/2021/200226

MS TITLE: SyNPL: Synthetic Notch pluripotent cell lines to monitor and manipulate cell interactions in vitro and in vivo.

AUTHORS: Mattias Malaguti, Rosa Portero Migueles, Jennifer Annoh Annoh, Daina Sadurska, Guillaume Blin, and Sally Lowell

I have now received all the referees' reports on the above manuscript, and have reached a decision. The referees' comments are appended below, or you can access them online: please go to BenchPress and click on the 'Manuscripts with Decisions' queue in the Author Area.

As you will see, the referees express considerable interest in your work, but have some significant criticisms and recommend a substantial revision of your manuscript before we can consider publication. Although Referee 1 is skeptical about the advance described in your study, I agree with Referees 2 and 3 that the reagents and method you have developed could be of interest to our readers as a Technique or Resource. I am therefore inviting a revision. I would ask you to respond to the points raised by Referees 2 and 3. In particular the issue of leakiness and the questions about the kinetics of the response seem important to address. While I agree with Referee 3 that analysing mouse lines made with this tool would be exciting, unless these data are already available, I don't think it is necessary. Nevertheless, timelapse imaging of blastocysts to test whether or not direct cell-cell interaction is necessary for receiver activation would greatly strengthen this conclusion.

If you are able to revise the manuscript along the lines suggested, which may involve further experiments, I will be happy receive a revised version of the manuscript. Your revised paper will be re-reviewed by one or more of the original referees, and acceptance of your manuscript will depend on your addressing satisfactorily the reviewers' major concerns. Please also note that Development will normally permit only one round of major revision.

We are aware that you may be experiencing disruption to the normal running of your lab that make experimental revisions challenging. If it would be helpful, we encourage you to contact us to

© 2022. Published by The Company of Biologists under the terms of the Creative Commons Attribution License (https://creativecommons.org/licenses/by/4.0/).

discuss your revision in greater detail. Please send us a point-by-point response indicating where you are able to address concerns raised (either experimentally or by changes to the text) and where you will not be able to do so within the normal timeframe of a revision. We will then provide further guidance. Please also note that we are happy to extend revision timeframes as necessary.

Please attend to all of the reviewers' comments and ensure that you clearly highlight all changes made in the revised manuscript. Please avoid using 'Tracked changes' in Word files as these are lost in PDF conversion. I should be grateful if you would also provide a point-by-point response detailing how you have dealt with the points raised by the reviewers in the 'Response to Reviewers' box. If you do not agree with any of their criticisms or suggestions please explain clearly why this is so.

Reviewer 1

Advance summary and potential significance to field

In this paper, Malaguti et al. describe the adaptation of the previously published SynNotch tool (Morsut et al. Cell, 2016) in mouse embryonic stem cells. The SynNotch technology, based on the re-engineering of the Notch-Delta signalling, exploits the cell-cell interaction between a “sender” and a “receiver” cell to drive a functional response of choice in the “receiver” cell.
A membrane-bound antigen on the surface of the “sender” cell is recognised by a synthetic Notch receptor on the surface of the “receiver” cell. Cell-cell contact thus triggers the activation of an intracellular effector domain in the “receiver” cell, leading to the expression of a gene of interest.
In order to establish clonal SynNotch pluripotent cell lines (SyNPL), Malaguti et al. applied a series of modifications to the original strategy (Morsut et al. 2016), among which: the use of electroporation rather than lentiviral infection the replacement of the promoters driving the expression of the components, the addition of HA and Myc tags to the sender (Delta) component, the use of a safe landing pad with recombination sites for easy substitution of the gene of interest in Rosa 26 locus.
The authors test the functionality of SynNotch in the derived clonal stem cells by measuring the kinetics of the induction using mCherry as the output gene. In addition, they interrogate the necessary time for cell-cell contact to trigger the response in receiver cells and the time required for mCherry signal to be lost.
Finally, Malaguti et al. demonstrate the functionality of SyNPL in vivo and in vitro. Chimaeric blastocysts were generated by mixing the same proportion of sender and receiver cells with a mouse morula and exhibited mCherry signal.
Moreover, spatial confinement was used to culture separately sender and receiver cells and upon confinement removal, a stripe pattern was formed in the region of contact between the two populations. By replacing the mCherry gene with Neurogenin 1, the authors were able to induce the differentiation of the patterned stripe into neurons.

Comments for the author

The results shown are well-founded, and SyNPL is a convenient tool for researchers who want to use SynNotch in ES cell-based models. However, the paper does not represent a sufficient technical/conceptual advance to justify publication. The modifications done to the original SynNotch system may facilitate its introduction and expression in mouse pluripotent stem cells, but they don’t alter, change or improve the functionality of the tool. Moreover while the HA and Myc tag modifications are functional, no clear application was specified in the text. Although the characterization done in figure 4 provides evidence that SynNotch is working in stem cells, it cannot be considered as the main finding since the kinetics of induction and required time of cell-cell contact had already been characterised by Morsut et al. (2016) in fibroblasts.
The chance of exploring the potentialities of SyNPL in figure 5 and 6 has been missed. Although the authors managed to obtain chimaeric SyNPL mouse embryos the tool wasn’t used to provide any new finding in blastocyst development. For figure 6, both pattern formation and SynNotch induced differentiation had been previously performed in fibroblasts by Morsut and colleagues. Altogether, the lack of new findings or the lack of significant technical improvement undermines the novelty of the paper, and I cannot recommend the publication of this paper in its current form.
In my opinion, the authors can either seek for a biological finding using the SyNPL or aim for publishing these results as a simple method paper. In the case of the second option, I would advise

© 2022. Published by The Company of Biologists under the terms of the Creative Commons Attribution License (https://creativecommons.org/licenses/by/4.0/).

Malaguti et al. to state in a clearer way the purpose of their experiments and to reduce the amount of details presented.
Comparing different strategies (e.g. the “all-in-one” design with the independent delivery of constructs in sender cells) is indeed valuable, but it could be reported in a more concise way.

Reviewer 2

Advance summary and potential significance to field

In this manuscript submitted as a resource/technical article, Malaguti and colleagues generate stable mES cell lines that are genetically engineered with synthetic signaling pathways of the synNotch family, both for sending synNotch signals and for receiving them. SynNotch pathways are used to engineer synthetic cell-cell communication pathways between ligand-producing sender cells, and receptor-expressing receiver cells.
To achieve this, the authors introduce genes for anti-GFP-synNotch receptors and for synNotch-inducible target genes (mCherry and Neurog1) alongside reporter genes to generate receiver cells; and genes for membrane-GFP to generate sender cells.

One major advance of this work is the demonstration of a very powerful way to introduce synNotch pathways in stem cells, which has not been reported before. SynNotch pathways have been used in a variety of cellular and in vivo contexts, but some reports indicated challenges with signal-to-noise ratio, prompting the development of new-generation receptors. The authors show here that some of the signal-to-noise issue could be addressed by carefully selecting promoters and genetic integration approaches to generate cell lines that respond with 100% activation to stimulation. These cell lines could be used in the field both to report on neighboring relationship for understanding neighboring relationship in developmental systems (organoids or embryos), and also, for forcing differentiation to control patterning and morphogenesis for enhancing control of organoids production.

Another advance in the paper is the quality of reporting both positive and negative "dead ends", and extensive primary data for a number of different clones, which, if it became the state of the art in the field, would substantially increase reproducibility and speed up progress.

The advancement of this paper lies in the authors' demonstration of a way to generate cell lines with highly controlled activation of synNotch pathways. These cell lines could have a widespread use in the community and could allow the reach of this novel synthetic biology tool outside the domain of strictly synthetic biology laboratories more into the developmental biology research.

Comments for the author

Couple of points that I would like to see resolved before publication:

One aspect that is not investigated is the leakiness of the selected clones; one question I have, and that would be good to know, is whether the leakiness in Fig. 3E is tetracycline-revertible? DAPT-sensitive? Would add one more information about this tool, for example, if this is to be used with target genes that require to have particularly low basal (e.g. suicide genes). I would also request that in Fig. 3E the real basal (with no transgenes) is included for reference. (The controls of Figs 3G and H are nice sanity checks, but seem more adequate as supplementary information in my opinion. The quasi-100% activation of Fig. 3F merit a figure just for themselves!!)
Do the authors see receiver cells becoming GFP+ in the FACS after contact with sender cells? Might be of interest for the community to know that; in some setups, receiver cells have been reported to become GFP+, which sometimes can make interpretation of results more complicated, especially when one bases results on separation of receivers from senders via the GFP channel in FACS.
Fig. 4 the exploration of the kinetics of the activation is interesting and speaks to one of the applications of the tool for monitoring cell-cell contacts; all their results here, as hinted by the authors, are highly dependent on the stability of the chosen reporter gene. It would be important to stress that in the presentation and discussion of the results. Target genes with a different half-life, or localization, for example, could have dramatically different dynamics of induction and degradation.

© 2022. Published by The Company of Biologists under the terms of the Creative Commons Attribution License (https://creativecommons.org/licenses/by/4.0/).

Fig. 5 The in vivo application is particularly exciting for the potential application. Some more characterization would increase the impact of this tool and inform others as of whether this is a tool that can be used for their experiments or not. I provide some suggestions on how to achieve that here:
Some more images of the other results shown in aggregate in the table would be great. Some questions that are not answerable with the data provided are:
Are there cases where not all the receiver cells are activated or always all receivers cells are activated? If that is the case, contact-dependency is hard to ascertain.
How was the "cherry-HI" label assigned? (automatically or manually? Was a threshold used?);
For supplementary images, of which I would like to request to see more to have a sense of the spread of what can be seen in these experiments in terms of activation intensity and robustness, and I would also request to have the colors split.
What were the dynamics of activation? How long after the aggregation do you see activation? Can the activation be prevented with DOX (or DAPT) for a certain time, and then induced at a later time point?
Fig. 6D The induction of functional transgenes is another highlight in terms of the potential application of the tool. Here too, more characterization would improve the impact of the description of the tool. Suggestions include:
What is the dynamic of activation of the transgene? And of the differentiation? Two fields of view are shown for this result, which is one of the most powerful potential uses of this tool (in my opinion); if the authors can give an idea of the robustness of this result it would increase impact: does it look like this in all the experiments? In all the fields of view? Is this a 100% activation like for the mCherry only?
The authors say that the receptors and the ligands are integrated into single copies according to references that used the same technology; would be important I think for the authors to confirm that in their clones, to answer the questions if receptors and ligands are integrated into single copies and if so where, for the 2 clones? It would be of interest to know, for the widespread use of the cell lines as tools, where the artificial constructs landed.

Minor points:
Fig. 2A/C is this supposed to have HA Somewhere?
"The CAG and mouse Pgk1 promoters are both silencing-resistant promoters commonly used to drive ubiquitous transgene expression in ESC" strong statement without reference
The authors do not explain, in the generation of eGFP+ sender cells, why they continued with mESCs expressing eGFP via CAG and not PGK.
"For both clones, the pattern of eGFP expression appeared to be consistent with membrane localization, and, in the case of CHmGMP19, eGFP co-localised with Myc (Fig. 2J,K)". This, to our reading, seems to imply that, for clone CmGP1, Myc-tag staining did not colocalize with eGFP. Is that the case? If so what do the authors think is the reason or plausible explanation?
Figure 3A. Why do the authors choose an anti-parallel ORF strategy for Pac and TRE-mCherry expression cassettes? This paper suggests that a sense orientation of transgenic cassettes with respect to rosa26 might lead to higher expression yields:
https://pubmed.ncbi.nlm.nih.gov/21853122/
The authors state "We verified that integration of the empty vector cassette led to loss of mKate2 expression, and used these control cell lines to confirm that tagBFP signal was able to unambiguously identify receiver cells (Figs. 3B,C, S11B)." but the loss of mKate2 expression is not shown in the quoted figures.
l.199; do you cultivate cells in presence of these antibodies?
Figure S11C-F. Numerical and statistical comparisons between the conventional TRE-mCherry and the extended tetO-mCherry outputs could improve the data presentation of these results. One way would be to include column bar charts to compare a) Flow cytometry mCherry MFI on receiver cells without senders b) Flow cytometry mCherry FMI on receivers co-cultured with senders and c) Fold-change or inducibility between monoculture and co-culture MFIs. Across these charts, each data point represents one clone, so that means and st.devs take into account clone-clone variation. This would allow readers to visually assess how TRE-mCherry and tetO-mCherry transgenes differ in terms of maximum output and inducibility.
Figure S12. Similarly, authors could consider increase data analysis to determine the numeric value for maximum fluorescence with respect to receiver:sender ratios. For example, an "Effective Ratio 50% (ER50)", which could be the % of sender cells in the co-culture required to activate 50% of

© 2022. Published by The Company of Biologists under the terms of the Creative Commons Attribution License (https://creativecommons.org/licenses/by/4.0/).

maximum mCherry fluorescence in receivers. This could be determined by fitting equation curves on mCherry MFI plotted against % of receivers.
Figure 4 D and Figure S13A: Considering tinitial = 0h and tfinal = 48h, what is the hour-mark that mCherry fluorescence reaches 50% of its maximum intensity?
Figure S12-13 are missing receivers co-cultures with wild-type or E14GIP1 “sender” negative controls.
Figure 6B: here too I have questions about the dynamics of the activation. Does the stripe of expression remain stable over time, or does cell locomotion and neighbor exchange cause the pattern to “diffuse” over time? If the latter, what is the time point at which the authors see the stripe forming, how long is it stable for, and when its “diffusion” is first evident? Why is that as a “gradient” from the zone of contact? Is there a lot of cell movement? This done with one clone? Other clones?
How reproducible is the result presented in one microscope picture?
One issue with the induction of differentiation is the epigenetic silencing: do the authors observe the loss of either receptor or trans gene or BFP while cultivating the mES cells in the pluripotent stage? What about after differentiation?
For the target gene, why do the authors think is a better approach to do recombinase integration as opposed to CRISPR in a specific locus? What is the advantage - if any?

Reviewer 3

Advance summary and potential significance to field

In this study, Lowell and colleagues adapt the Synthetic Notch (SynNotch) system to mouse embryonic stem cells (mESCs) as a tool for investigating cell-cell contacts.

The SynNotch system was first described in 2016. It exploits direct cell-cell contact as a trigger to control custom downstream effects, for example the expression of a certain genetic cassette (e.g. reporters as live imaging read-outs). In this way, cells can be engineered to express reporters of interest in response to physical contact between two neighboring cells mediated by engagement of Notch signaling interactions. This system has already been applied in various contexts.

In this manuscript, the authors report engineering mESCs to constitutively express components of the SynNotch system, and the subsequent validation of the system (predominantly in vitro). They produce clonal cell lines that act as either “sender” or “receiver” cells. Their “receiver” cells have a modular design, such that they can be further engineered to introduce any cassette of interest who’s expression would be regulated in reponse to a cell-cell contact (i.e. downstream of Notch signaling). The authors convincingly demonstrate the activity of the system in vitro in mESCs (mixed cultures and multi-well plated) and in vivo in mouse blastocyst stage embryo chimeras containing their engineered mESCs.

Overall, while the technical novelty of this study may be limited, the mESC lines generated by the authors are likely to be a useful resource for the community.
However, the availability of equivalent mice derived from the mESCs generated here would have been even more desirable for the community.

Comments for the author

Major comments:
1. It would have been valuable had the authors assessed the system and their mESCs in post-implantation embryo chimeras. How effective would it be to generate ‘dual’ chimeras by introducing 2 types of mESCs into wild-type embryo hosts and analyzing them after embryo implantation (i.e. beyond the blastocyst stage)?
Namely how does the developmental potential of the “sender” vs. “receiver” mESC lines generated in this study compare, and are they developmentally neutral? This is important to know should anyone want to further engineer the “receiver” cells to their own design and then make a mouse..
2. It is disappointing the authors did not make mice out of their mESCs which could then be aggregated at the 8-cell stage to generate more efficient chimeras of ‘sender’ and ‘receiver’ cells (negating the above point). This would be important for the general utility of the system and its

© 2022. Published by The Company of Biologists under the terms of the Creative Commons Attribution License (https://creativecommons.org/licenses/by/4.0/).

application to different contexts, beyond ESC co-cultures and blastocyst stage embryo ICMs. For example, were the parental "receiver' cells not able to make a mouse, then any downtream studies someone might plan will be limited to in vitro work. If a mouse is not generated, this caveat of the system should be discussed.
3. For such an mESC resource the authors have disappointingly limited themselves to just using parental E14 mESCs.
4. Blastocyst validation: these results suggest that the system is functional, but it is difficult to prove that direct cell-cell interaction was required. Perhaps a time-lapse movie post-chimerism with higher receiver : sender cell ratios (see point 1) would better demonstrate this point. Additionally, a discussion of various scenarios of how this system, given its kinetics, could be helpful in blastocyst development would be useful.
5. The discussion concerning cell competition at the end of the paper is not clear and may not be practical: how might a 'losing' sender cell would be defined from a 'non-losing' sender cell? How could one separate the receivers from either?
If receiver and sender cells come in contact during the generation of embryo chimeras, could that add noise to the system? Perhaps cell lineage specific drivers, coupled with Dox control, may be more appropriate?
6. The authors should comment on the availability, deposition in repositories and distribution of the generated lines.

Minor/editorial comments:
1. Figure 1: Present an overview of Notch signaling to place this tool into context. At present this is hard to understand for the non specialists.
2. Figure 1: label the nucleus vs. cytoplasmic compartment at least in one of the cell diagrams.
3. Line 101 – 104, it is still possible to get multi-copy integration with electroporation. Other methods (southern blot or sequencing) would be needed to ensure the integration of a single copy.
4. Line 116. Unclear what the purpose of the HA/Myc tag on the EGFP is? A brief description is provided later, but does not clarify what can be achieved with these tags that cannot be achieved with EGFP (e.g. sorting using direct fluorescence or indirectly using antibodies).
5. Please change eGFP to EGFP (current correct nomenclature?).
6. Line 128. Did the authors also stain for HA?
7. Line 245-265: Quantification of induction dynamics. The authors explore kinetics using time-lapse imaging, and overall population changes using FACS.
These two approaches are complimentary, but perhaps the imaging is more accurate because it is inherently at single-cell resolution which is important in this context. However, the authors do not provide quantifications, statistics, or detailed methodology of how they reached their imaging-based conclusions. Please provide how many cells were tracked, the variance in induction dynamics, the tracking method (manual, semi-automated or fully automated).
8. Line 275-279: Given the authors' data, two factors can affect the reporter (mCherry) signal intensity: (1) duration of the contact, (2) time lapsed since the contact. Thus, a weak signal could mean one of three possibilities: (1) a long interaction that ended many hours ago, (2) a short recent interaction that just ended, or (3) a newly established interaction. Presumably it would not be possible for the system to discriminate between these possibilities. Please clarify how this might be possible or include this as a caveat of the system.

9. Line 323: Harnessing the system for cell fate induction. These results are exciting. Given the increasing body of literature demonstrating that mechanics impact signaling and can drive mESC fate decisions, it may be important to show a control for this experiment using a normal non-membrane GFP to prove that the mechanical strain at the border of these two populations is not driving the observed fate change.

© 2022. Published by The Company of Biologists under the terms of the Creative Commons Attribution License (https://creativecommons.org/licenses/by/4.0/).

First revision

Author response to reviewers' comments

We do not provide a response to Reviewer 1 because we were asked by the Editor to respond only to the concerns of Reviewers 2 and 3.

Reviewer 2

Advance Summary and Potential Significance to Field:

In this manuscript submitted as a resource/technical article, Malaguti and colleagues generate stable mES cell lines that are genetically engineered with synthetic signaling pathways of the synNotch family, both for sending synNotch signals and for receiving them. SynNotch pathways are used to engineer synthetic cell-cell communication pathways between ligand-producing sender cells, and receptor-expressing receiver cells. To achieve this, the authors introduce genes for anti-GFP-synNotch receptors and for synNotch-inducible target genes (mCherry and Neurog1) alongside reporter genes to generate receiver cells; and genes for membrane-GFP to generate sender cells.

One major advance of this work is the demonstration of a very powerful way to introduce synNotch pathways in stem cells, which has not been reported before. SynNotch pathways have been used in a variety of cellular and in vivo contexts, but some reports indicated challenges with signal-to-noise ratio, prompting the development of new-generation receptors. The authors show here that some of the signal-to-noise issue could be addressed by carefully selecting promoters and genetic integration approaches to generate cell lines that respond with 100% activation to stimulation.

These cell lines could be used in the field both to report on neighboring relationship for understanding neighboring relationship in developmental systems (organoids or embryos), and also, for forcing differentiation to control patterning and morphogenesis for enhancing control of organoids production.

Another advance in the paper is the quality of reporting both positive and negative "dead ends", and extensive primary data for a number of different clones, which, if it became the state of the art in the field, would substantially increase reproducibility and speed up progress.

The advancement of this paper lies in the authors' demonstration of a way to generate cell lines with highly controlled activation of synNotch pathways. These cell lines could have a widespread use in the community and could allow the reach of this novel synthetic biology tool outside the domain of strictly synthetic biology laboratories more into the developmental biology research.

Reviewer 2 Comments for the Author:

Couple of points that I would like to see resolved before publication:

[2.1] One aspect that is not investigated is the leakiness of the selected clones; one question I have, and that would be good to know, is whether the leakiness in Fig. 3E is tetracycline-revertible? DAPT-sensitive? Would add one more information about this tool, for example, if this is to be used with target genes that require to have particularly low basal (e.g. suicide genes).

Thanks for this great suggestion. We address this with new data (Supp Fig. 12B-G). The leakiness observed in receiver cells cultured alone can be reduced, but not completely abolished, by treatment with doxycyline or DAPT for 48h. This residual leakiness is unlikely to be a problem for most applications, but we agree that there are exceptions where leakiness may matter so it is important to make readers aware of this.

[2.2] I would also request that in Fig. 3E the real basal (with no transgenes) is included for reference. (The controls of Figs 3G and H are nice sanity checks, but seem more adequate as supplementary information in my opinion. The quasi-100% activation of Fig. 3F merit a figure just for themselves!!)

The basal expression with no transgenes is shown in Fig. 3B,C, but we realise that we did not make it clear that this data could be directly compared with the data in Fig. 3E. We have now clarified this in the figure legend.

© 2022. Published by The Company of Biologists under the terms of the Creative Commons Attribution License (https://creativecommons.org/licenses/by/4.0/).

[2.3] Do the authors see receiver cells becoming GFP+ in the FACS after contact with sender cells? Might be of interest for the community to know that; in some setups, receiver cells have been reported to become GFP+, which sometimes can make interpretation of results more complicated, especially when one bases results on separation of receivers from senders via the GFP channel in FACS.

Yes, we do see receiver cells becoming weakly GFP+ after contact with sender cells (new data in Fig. S12B). Punctate GFP signal in receivers indicates that small amounts of GFP are being internalised into receivers rather than sticking to the cell surface (new data in Fig. S12C). This highlights the value of the tagBFP lineage label that we introduced into receiver cells: this allows us to to unambiguously identify receiver cells even if they are weakly GFP+ (Fig. S12E). It also highlights the value of our CmGP1GH1 sender line, which contains an extra EGFP transgene, boosting separation between senders and receivers in the EGFP channel (Fig. S12F,G).
We do not believe that GFP cross-labelling interferes with the utility of the system, but agree that this information is useful to the community.

[2.4] Fig. 4 The exploration of the kinetics of the activation is interesting and speaks to one of the applications of the tool for monitoring cell-cell contacts; all their results here, as hinted by the authors, are highly dependent on the stability of the chosen reporter gene. It would be important to stress that in the presentation and discussion of the results. Target genes with a different half-life, or localization, for example, could have dramatically different dynamics of induction and degradation.

We agree. Our discussion includes the following sentence.
Text highlighted in discussion :"Should this persistence of mCherry signal prove inconvenient for the study of particular processes, the PSNB landing pad parental cell lines can be used to readily generate cell interaction reporter receiver cells harbouring destabilised inducible transgenes with short half-lives."

We are already more than 1000 words above our word limit so we hope that this will be sufficient to convey the point raised by this reviewer, even though it would be nice to discuss this in more depth (i.e. not just in relation to fluorescent proteins) if we had the space.

[2.5] Fig. 5 The in vivo application is particularly exciting for the potential application. Some more characterization would increase the impact of this tool and inform others as of whether this is a tool that can be used for their experiments or not. I provide some suggestions on how to achieve that here:
Some more images of the other results shown in aggregate in the table would be great.

We provide more images in new Fig. S18A

[2.6] Some questions that are not answerable with the data provided are:
Are there cases where not all the receiver cells are activated or always all receivers cells are activated? If that is the case, contact-dependency is hard to ascertain.

We provide new data (Fig. S18B) to show that there are indeed cases where not all BFP+ receiver cells are activated. Extensive neighbour-exchange occurs in the inner cell mass of preimplantation embryos (Plusa et al, Development 2008) making it challenging to create a situation where receivers are unlikely to have significant encounters with senders, but we are pleased to report that did manage to achieve this by aggregating morulae with a single sender cell along with approximately 8 receiver cells, which were delivered to the opposite side of the morulae from the single sender (we are lucky that our embryologist co-author has such impressive skills!). In this context, some of the BFP+ receiver cells remain negative for mCherry even at the blastocyst stage.

In addition to this, we also now have additional data supporting our finding that receiver cells within chimeric blastocysts do not express mCherry in the absence of any sender cells (update to number shown in the table in Fig. 6B).

© 2022. Published by The Company of Biologists under the terms of the Creative Commons Attribution License (https://creativecommons.org/licenses/by/4.0/).

[2.7] How was the “cherry-HI” label assigned? (automatically or manually? Was a threshold used?);

Scoring of mCherry-HI cells was performed manually using chimaeras containing both sender and receiver cells as a reference. The Cherry signal was readily and unambiguously detectable in all cases.
We provide this information in the legend of Fig.6 (highlighted text) and in the Materials and Methods (Highlighted text in ‘Immunofluorescence’ section.)

[2.8] For supplementary images, of which I would like to request to see more to have a sense of the spread of what can be seen in these experiments in terms of activation intensity and robustness, and I would also request to have the colors split.

We provide these data in Fig. S18A. The labelling worked robustly in all experiments and although it is not appropriate to directly compare signal intensity between different experiments, the mCherry signal was clearly detectable in all cases.

2.8 What were the dynamics of activation? How long after the aggregation do you see activation?

The earliest time points that we see clear activation is generally between 4 and 6 hours, although there is considerable variation (up to 20h: see new supplemental Movie 6), most likely relating to variability in the time taken for sender cells to first encounter receiver cells.

It would have been interesting to also directly track sender and receiver cells from the start of aggregation in live embryos in order to measure time of contact in each embryo, but this is not possible due to UV toxicity when live-imaging BFP. Please see response to reviewer 3 point 3.4 for a summary of our extensive efforts to try to overcome this problem, which ultimately proved insurmountable.

[2.9] Can the activation be prevented with DOX (or DAPT) for a certain time, and then induced at a later time point?

Yes. These new data are shown in new Fig. S17 (using DAPT)

[2.10] Fig. 6D

The induction of functional transgenes is another highlight in terms of the potential application of the tool. Here too, more characterization would improve the impact of the description of the tool. Suggestions include:
What is the dynamic of activation of the transgene? And of the differentiation?

We provide a new figure (Fig. S21B) showing daily time points over the course of 6 days. 3xFlag-Neurog1 is first detected 2 days after insert removal, corresponding to less than 24 hours after the two cell populations come into direct contact. This is in keeping with the dynamics of mCherry transgene induction in our other experiments: Neuronal differentiation marker Tubb3 is first seen on day 4 and persists for at least 96 hours after initial contact between the sender and receiver cells (we could not try longer time points because cultures would become excessively over-confluent).

[2.11] Two fields of view are shown for this result, which is one of the most powerful potential uses of this tool (in my opinion); if the authors can give an idea of the robustness of this result it would increase impact: does it look like this in all the experiments? In all the fields of view? Is this a 100% activation like for the mCherry only?

Induction of neurons by Neurog1 occurs at low efficiency in pluripotent cells under pluripotency-maintenance conditions, even when expressed from a conventional plasmid using a high-efficiency promoter such as CAG (our own observations and, for example, Figure 1 of doi: 10.1371/journal.pone.0038651). Furthermore, Neurog1 stability is regulated post-transcriptionally, creating variability in expression even from a constitutive promoter (Our observations and, for example, doi: 10.1042/BJ20070064.). For both these reasons, we did not

© 2022. Published by The Company of Biologists under the terms of the Creative Commons Attribution License (https://creativecommons.org/licenses/by/4.0/).

expect a high efficiency induction of neurons in our 'stripe' experiment. We do however see induction of neurons in every experiment and in most, although not all, fields of view.

We provide a new figure (Fig. S21B) that shows Neurog1 transgene expression and expression of neuronal differentiation marker TUBB3 at several time points. We show three fields of view in each case to illustrate typical variability between fields of view in these experiments.

[2.12] The authors say that the receptors and the ligands are integrated into single copies according to references that used the same technology; would be important I think for the authors to confirm that in their clones, to answer the questions if receptors and ligands are integrated into single copies and if so where, for the 2 clones? It would be of interest to know, for the widespread use of the cell lines as tools, where the artificial constructs landed.

We have removed the suggestion that transgenes are likely to be single copy inserts. We no longer have the facilities in our institute to perform Southern blots, and hope that the reviewer agrees that this is not a critical point.
We also note that it be straightforward to apply the SyNPL system to any mouse ES cell line of choice using our plasmids and optimised strategies, so it is likely that at least some users will in any case take this option rather than using our original cell lines.

Minor points:

[2.13] Fig. 2A/C is this supposed to have HA Somewhere?

No, these are the untagged sender cell lines

[2.14] "The CAG and mouse Pgk1 promoters are both silencing-resistant promoters commonly used to drive ubiquitous transgene expression in ESC"strong statement without reference

Thank you for picking up this omission. References have now been added.

[2.15] The authors do not explain, in the generation of eGFP+ sender cells, why they continued with mESCs expressing eGFP via CAG and not PGK.

There was no strong reason for preferring one promoter over the other: sender cells worked well in both cases. There is perhaps a slightly tighter distribution of GFP when using the CAG promoter (Figs S1 and S2), but otherwise no particular advantage that we know of over PGK. If the reviewer thinks it useful to add a sentence to the text to this effect then we would be happy to do so.

[2.16] "For both clones, the pattern of eGFP expression appeared to be consistent with membrane localization, and, in the case of CHmGMP19, eGFP co-localised with Myc (Fig. 2J,K)". This, to our reading, seems to imply that, for clone CmGP1, Myc-tag staining did not colocalize with eGFP. Is that the case? If so what do the authors think is the reason or plausible explanation?

Clone CmGP1 is not tagged with Myc. We used both tagged and untagged versions of the sender construct and did not see any difference in sender activity (Fig. S13). We realise this is confusing so have modified the text as follows:
"For both clones, the pattern of EGFP expression appeared to be consistent with membrane localisation, and, in the case of the HA- and Myc-tagged CHmGMP19 clone, the pattern of HA and Myc expression appeared to coincide with that of EGFP (Fig. 2J)."

[2.17] Figure 3A. Why do the authors choose an anti-parallel ORF strategy for Pac and TRE-mCherry expression cassettes? This paper suggests that a sense orientation of transgenic cassettes with respect to rosa26 might lead to higher expression yields: https://pubmed.ncbi.nlm.nih.gov/21853122/

© 2022. Published by The Company of Biologists under the terms of the Creative Commons Attribution License (https://creativecommons.org/licenses/by/4.0/).

For TRE-mCherry we were aiming for tight regulation rather than high expression yield, so we were not concerned about choosing the orientation that would give higher expression in this case, and had no reason to prefer one orientation over the other. Pac (i.e. PuroR) is in the sense orientation (and driven by the Rosa26 promoter.)

[2.18] The authors state “We verified that integration of the empty vector cassette led to loss of mKate2 expression, and used these control cell lines to confirm that tagBFP signal was able to unambiguously identify receiver cells (Figs. 3B,C, S11B).” but the loss of mKate2 expression is not shown in the quoted figures.

Apologies for not including this data in our original manuscript. Fig. S11B now contains panels displaying mKate2 levels in PSNB-A and PSNB-B parental lines, and panels displaying loss of mKate2 levels in PSNBA-E and PSNBB-E empty vector lines.

[2.19] l.199; do you cultivate cells in presence of these antibodies (antibiotics?)?

Yes. This information is provided in the methods (highlighted text in the “ESC culture” section).

[2.20] Figure S11C-F. Numerical and statistical comparisons between the conventional TRE-mCherry and the extended tetO-mCherry outputs could improve the data presentation of these results. One way would be to include column bar charts to compare
a) Flow cytometry mCherry MFI on receiver cells without senders b) Flow cytometry mCherry FMI on receivers co-cultured with senders and c) Fold-change or inducibility between monoculture and co-culture MFIs. Across these charts, each data point represents one clone, so that means and st.devs take into account clone-clone variation. This would allow readers to visually assess how TRE-mCherry and tetO-mCherry transgenes differ in terms of maximum output and inducibility.

Thank you for this suggestion. Please see new panel G in Fig. S11. This way of plotting the data does indeed make it easier to visually compare the performance of TRE vs tetO.

[2.21] Figure S12. Similarly, authors could consider increase data analysis to determine the numeric value for maximum fluorescence with respect to receiver:sender ratios. For example, an “Effective Ratio 50% (ER50)”, which could be the % of sender cells in the co-culture required to activate 50% of maximum mCherry fluorescence in receivers. This could be determined by fitting equation curves on mCherry MFI plotted against % of receivers.

Thank you for this suggestion. After some consideration we would prefer not to present the data in this way because our focus here is to assess the proportion of cells that activate maximum fluorescence within a bimodal distribution of ‘responders’ vs ‘non-responders’ (Fig. S13C-H, previously Fig. S12C-H). In this context we don’t feel the relative degree of activation provides readily interpretable information.

[2.22] Figure 4 D and Figure S13A: Considering tinitial = 0h and tfinal = 48h, what is the hour-mark that mCherry fluorescence reaches 50% of its maximum intensity?

Our original flow cytometry data (Fig. 4D and S14B - previously S13A) could only narrow this down to a time after 8h but before 24h, so we have now performed new live cell imaging experiments in which we measure fluorescence in individual cells every hour between 0h and 24h. This shows that the time point at which mCherry fluorescence reaches 50% of its intensity at 24h is approximately 15h. We provide these data in new Figs 4F,G, S14C-H.

[2.23] Figure S12-13 are missing receivers co-cultures with wild-type or E14GIP1 “sender” negative controls.

We provide new data in new Fig. 4F and Fig. S14C-E to show that E14GIP1 “fake sender” cells are not able to induce mCherry. This confirms the control data from the 24h time point, shown in Fig. 3G. We have not also added WT or E14GIP1 cells to the experiments shown in Figs S12 or S13: it doesn’t seem necessary to further confirm the very clear control data that we show in Figs 3, 4F and S14C-E, plus it would be technically extremely challenging (if not impossible) to add even more cell lines to these enormous experiments, which are already at the limit of the number of

© 2022. Published by The Company of Biologists under the terms of the Creative Commons Attribution License (https://creativecommons.org/licenses/by/4.0/).

samples than can be realistically processed within the necessary timespan.

[2.24] Figure 6B: here too I have questions about the dynamics of the activation. Does the stripe of expression remain stable over time, or does cell locomotion and neighbor exchange cause the pattern to "diffuse" over time?

The pattern does seem to diffuse slightly over time. We have collected new data, taking images from 2 different clones, each at sixteen regular time points from 24h after insert removal (i.e before the mCherry+ red stripe first becomes detectable) up to 118h. The stripe generally remains distinct but over time a few red cells do appear within the green domain rather than being strictly restricted to the boundary (see FigS19A and discussion within supplementary methods)

Examining imaging in the z plane shows that green sender cells seem to sometimes migrate on top of receiver cells (Fig. S19C) and to infiltrate into the blue receiver zone (Fig S19B), likely contributing to the 'diffusion' effect. There is considerable proliferation during this period, so cell division likely also contributes to the 'diffusion' effect (Fig. S19B).

In summary, the stripe remains stable over several days, with a slight diffusion effect that is likely due to a combination of cell migration and cell division.

[2.25] If the latter, what is the time point at which the authors see the stripe forming, how long is it stable for, and when its "diffusion" is first evident?

This varies somewhat between experiments, but initial contact is made approximately two days after insert removal, and mCherry induction is observed several hours after that, similar to the timing of Neurog1 induction shown in new Fig. S21B.

The stripe persists in culture for at least three days. We have not tested this for longer time points: cell death becomes more prominent the longer cells are kept in culture without passaging.

"Diffusion" of the stripe is evident from the time of onset of mCherry expression. As discussed above, it seems likely that cell division, neighbour exchange, and sender cell migration all play a part in the diffusion of the pattern.

[2.26] Why is that as a "gradient" from the zone of contact

We can't say for sure, but it seems likely that this "gradient" appears because cells further from the border may have had a shorter contact time with receivers. This is speculation so we do not discuss this in our paper.

[2.27] Is there a lot of cell movement?

We attempted to address this using time lapse imaging, but in order to track individual cells we needed to film at short time intervals, and this unfortunately led to rapid bleaching of fluorescence. There is a lot of cell division that makes it difficult to assess exactly how much cells move in general based on time-lapse of phase-contrast images, but there is certainly some cell movement.

[2.28] This done with one clone? Other clones? How reproducible is the result presented in one microscope picture?

The "stripe" result is reproducible across all three clones and all replicates. Our supplemental material is already rather bloated so have not included data from all three clones in our current manuscript (the supplemental figure shows data from two clones) but would be happy to make new supplemental figures to show these data if the reviewer thinks this is important.

© 2022. Published by The Company of Biologists under the terms of the Creative Commons Attribution License (https://creativecommons.org/licenses/by/4.0/).

[2.29] One issue with the induction of differentiation is the epigenetic silencing: do the authors observe the loss of either receptor or trans gene or BFP while cultivating the mES cells in the pluripotent stage? What about after differentiation?

We have never observed silencing of receptor, BFP or GFP expression, nor of transgene-inducibility, over the many experiments we have performed over more than two years with these cell lines under pluripotency conditions.

We provide new data (Fig. S18C) confirming that BFP, GFP and mCherry can all be readily detected extensively throughout high-contribution post-gastrulation chimeric embryos, indicating that the system remains functional in differentiated cells throughout the embryo.

We have not performed an exhaustive characterisation of multiple differentiated cell types in monolayer culture, but we can report that the system continues to work well in differentiated cells (5 days of LIF withdrawal) in all three clones when selection is maintained, with some clone-dependent variability in the absence of selection. We see no evidence of silencing of either BFP or GFP after 5 days of LIF-withdrawal (see Figure R1 below).

The post-implantation chimera data gives the strongest evidence that the system works robustly in multiple differentiated cell types and so we include that data, but not the more limited LIF-withdrawal data, in our revised manuscript.

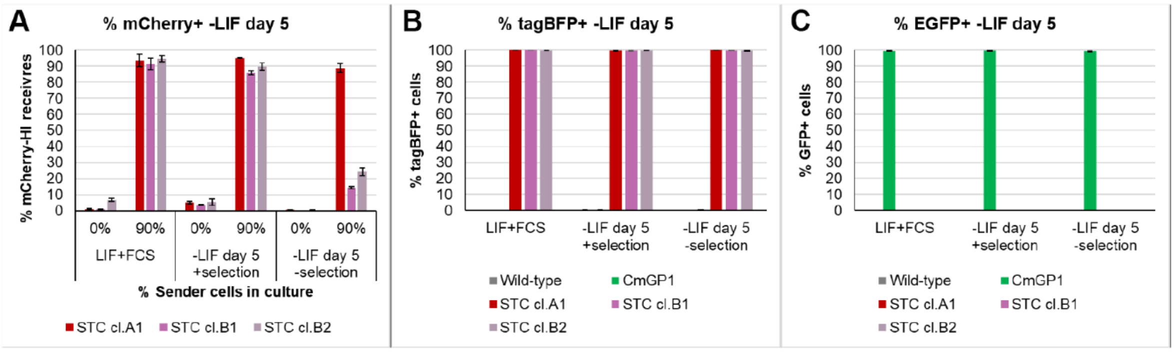

Figure R1: mCherry inducibility and transgene persistence in LIF withdrawal.
(A) Flow cytometry analysis of mCherry expression in STC clone A1, B1 or B2 receiver cells cultured in pluripotency conditions (LIF+FCS) or subject to LIF withdrawal either in the presence or in the absence of selective antibiotics for 4 days. Cells were passaged on day 2 and day 4. On day 4, cells were replated either alone or with CmGP1 sender cells (cultured in the same conditions) for a further 24 hours, before being assayed by flow cytometry.
(B-C) Flow cytometry analysis of (B) tagBFP and (C) EGFP expression in wild-type (E14Ju09), CmGP1 sender cells, and STC clone A1, B1 and B2 receiver cells cultured alone in pluripotency conditions (LIF+FCS) or in LIF withdrawal medium either in the presence or in the absence of selective antibiotics for 5 days. Cells were passaged on day 2 and day 4.

[2.30] For the target gene, why do the authors think is a better approach to do recombinase integration as opposed to CRISPR in a specific locus? What is the advantage - if any?

We prefer this approach because we believe it is faster and more convenient and carries less risk of off-target genome editing.

Reviewer 3
Advance Summary and Potential Significance to Field:

In this study, Lowell and colleagues adapt the Synthetic Notch (SynNotch) system to mouse embryonic stem cells (mESCs) as a tool for investigating cell-cell contacts.

The SynNotch system was first described in 2016. It exploits direct cell-cell contact as a trigger to control custom downstream effects, for example the expression of a certain genetic cassette

© 2022. Published by The Company of Biologists under the terms of the Creative Commons Attribution License (https://creativecommons.org/licenses/by/4.0/).

(e.g. reporters as live imaging readouts). In this way, cells can be engineered to express reporters of interest in response to physical contact between two neighboring cells mediated by engagement of Notch signaling interactions. This system h as already been applied in various contexts.

In this manuscript, the authors report engineering mESCs to constitutively express components of the SynNotch system, and the subsequent validation of the system (predominantly in vitro). They produce clonal cell lines that act as either “sender” or “receiver” cells. Their “receiver” cells have a modular design, such that they can be further engineered to introduce any cassette of interest who’s expression would be regulated in reponse to a cell-cell contact (i.e. downstream of Notch signaling). The authors convincingly demonstrate the activity of the system in vitro in mESCs (mixed cultures and multi-well plated) and in vivo in mouse blastocyst stage embryo chimeras containing their engineered mESCs.

Overall, while the technical novelty of this study may be limited, the mESC lines generated by the authors are likely to be a useful resource for the community. However, the availability of equivalent mice derived from the mESCs generated here would have been even more desirable for the community.

Reviewer 3 Comments for the Author:
Major comments:

[3.1].It would have been valuable had the authors assessed the system and their mESCs in post-implantation embryo chimeras. How effective would it be to generate ‘dual’ chimeras by introducing 2 types of mESCs into wild-type embryo hosts and analyzing them after embryo implantation (i.e. beyond the blastocyst stage)?

Namely how does the developmental potential of the “sender” vs. “receiver” mESC lines generated in this study compare, and are they developmentally neutral? This is important to know should anyone want to further engineer the “receiver” cells to their own design and then make a mouse..

Thank you for this excellent suggestion. We have now generated high-contribution dual chimaeras dissected at gastrulation and early post-gastrulation stages. These new data are shown in Fig. S18C. Both the sender and receiver cells are able to contribute extensively throughout the embryos, indicating that they retain developmental potency.

Most importantly, we detect extensive mCherry labelling, indicating that the system remains functional throughout post-implantation embryos.

[3.2]
[2.It]2.It is disappointing the authors did not make mice out of their mESCs, which could then be aggregated at the 8-cell stage to generate more efficient chimeras of ‘sender’ and ‘receiver’ cells (negating the above point). This would be important for the general utility of the system and its application to different contexts, beyond ESC co-cultures and blastocyst stage embryo ICMs. For example, were the parental “receiver’ cells not able to make a mouse, then any downtream studies someone might plan will be limited to in vitro work. If a mouse is not generated, this caveat of the system should be discussed.

We agree it could be interesting to generate a ‘receiver’ mouse line to use to generate host blastocysts as an alternative to our ‘dual chimaera’ strategy. It would also be interesting to graft sender cells into post-implantation ‘receiver’ embryos to study how the grafted cells influence their local neighbourhood. This is beyond the scope of our own work and we have not included this point in our revised discussion because we are already well over the word limit for our paper. We hope this is acceptable to the reviewer and editor.

[3.3].For such an mESC resource the authors have disappointingly limited themselves to just using parental E14 mESCs.

© 2022. Published by The Company of Biologists under the terms of the Creative Commons Attribution License (https://creativecommons.org/licenses/by/4.0/).

Our DNA constructs and extensive characterisation will make it possible for any researcher to apply this system to any ES cell line of their choice.

[3.4].Blastocyst validation: these results suggest that the system is functional, but it is difficult to prove that direct cell-cell interaction was required. Perhaps a time-lapse movie post-chimerism with higher receiver : sender cell ratios (see point 1) would better demonstrate this point.
To our knowledge nobody has ever reported three-colour time-lapse imaging of the morula to blastocyst transition. Cells undergoing this transition are particularly susceptible to UV light, greatly limiting the opportunity to regularly image BFP (our marker of receiver cells) without killing the cells. Furthermore, there is frequent neighbour-exchange within the inner cell mass of blastocysts (Plusa et al Development 2008), so cells can only be tracked over time if imaged at very frequent intervals (and even then, with some difficulty given that these are 3D structures).

Nevertheless, we were excited to take on this challenge and tried very hard to find work-arounds to the problems we lay out above. Some of the things we tried are listed at the end of our response to this point

To cut a long story short: after some optimisation, we were successful in filming GFP and capturing mCherry induction within preimplantation embryos in several experiments. This has allowed us to address the question of how long after aggregation mCherry can generally be detected: see response 2.8 to reviewer 2 and new Movie 6.

Unfortunately (although not surprisingly) it proved impossible to directly track interactions between BFP+ receivers and GFP+ senders in this highly dynamic and UV-sensitive context, so we could not address the question of exactly how long receiver cells needed to directly contact sender cells before the mCherry signal appeared.

We have, however, performed several other new experiments that argue against a non-specific contact-independent activation of mCherry in embryos:
1) *When sender-cell numbers are limiting, not all receiver cells activate mCherry in embryos (new Fig. S18B, see point 2.6 to reviewer 2)*
2) *DAPT, which interferes with contact-dependent cleavage of the SynNotch receptor, suppresses mCherry induction (new Fig. S17 see point 2.9 to reviewer 2)*
3) *We have increased the number of control aggregations with receiver cells but no sender cells. This confirms that mCherry induction depends on the presence of sender cells (although we acknowledge that this does not prove dependence on direct contact). Updated data in Fig. 6B, see point 2.6 to reviewer 2.*

In summary: *the new data that we have provided gives confidence that there is unlikely to be any unexpected sender-independent labelling when the SyNPL system is used in vivo, even though we were not able to reliably live-image all contacts between sender cells and receiver cells in embryos.*

For information, below we list the various strategies used to (unsuccessfully) attempt to capture all sender-receiver interactions within preimplantation embryos. In all cases, morulae were aggregated with GFP+ sender ES cells and BFP+ receiver ES cells.

1) *Imaging GFP BFP & mCherry at 10 min intervals from the morula stage. BFP bleached to undetectable levels after only 1h 20 mins, embryos died after 3h*

2) *Similar to (1), but imaging at 1h intervals. Embryos died after 14h, BFP had bleached by this time.*

3) *Similar to (2), but starting from the early blastocyst stage (24 hours post-aggregation), imaging at 2h intervals and only imaging BFP in the first and last frame. mCherry was present in receiver cells from the start of the movie, and increased over time. The embryos survived. We attempted to manually track the descendants of the original BFP+ cells in phase contrast images so that we could see when BFP+ cells contacted GFP+ cells, but this proved impossible due to the extensive*

© 2022. Published by The Company of Biologists under the terms of the Creative Commons Attribution License (https://creativecommons.org/licenses/by/4.0/).

cell movements and cell divisions in these 3D structures. Furthermore, the extensive neighbour-exchange meant that we would in any case miss any cell-cell interactions that occurred between the two-hourly images. So, although it was possible to capture mCherry induction in some cells, we could not know if or when these cells had previously contacted sender cells.

4) *Similar to (3) but starting from the morula stage using 2h intervals. Again, it proved impossible to track the descendants of the BFP+ sender cells, and embryos died around 22-24h, but it was possible to capture mCherry induction in some cells*

5) *Starting from the blastocyst stage 24h post aggregation and filming for a short period at 15 min intervals in a last-ditch attempt to track cells at least for a while, hoping to capture sender-receiver interactions. Spy650 nuclear-labelling dye was used to identify GFP- receiver cells given that it was not possible to image BFP in multiple frames without causing cell death. Again, we saw some cell death, and found it impossible to unambiguously track receiver cells even using Spy650, but again we did manage to capture mCherry induction in some cells.*

6) *Starting filming 4h after aggregation, 30 minute intervals. First frame included BFP imaging. We cultured some embryos in the presence of Spy650. We hoped this might at last give us a chance of accurately tracking BFP+ Spy650+ receiver cells. Embryos cultured with Spy650 died rapidly, hence tracking of cells was still not possible. Most embryos cultured without Spy650 also died, but we did manage to capture mCherry induction in some cells in embryos which survived.*

[3.4b] Additionally, a discussion of various scenarios of how this system, given its kinetics, could be helpful in blastocyst development would be useful.

We envisage that this system could be helpful for testing hypotheses about molecular interactions that mediate lateral inhibition during specification of PE and EPI (eg Simon et al 2018 doi: 10.1002/wdev.319) or physical cell sorting of PE from EPI (Plusa at al doi: 10.1242/dev.021519) or lateral induction to stabilise locally coherent fates once PE and EPI have segregated, either during normal development or during diapause (something that, to our knowledge,has not yet been explored). These things should be possible to address using SyNPL given that some of the signals and TFs that autonomously drive cell fates are known and could be engineered into sender cells in order to discover how cell-autonomous differentiation impacts on the fate or position of neighbouring cells. . All of the events we describe above seem to operate over many hours (as far as can be determined) and so there is a good chance that these types of experiments would be suited to the kinetics of our system.

We are concerned that our paper is becoming over-long for a Techniques and Resources article, and we are already more than 1000 words over the word limit, so we prefer not to include these ideas in our discussion, if this is acceptable to the reviewer and editor.

[3.5].The discussion concerning cell competition at the end of the paper is not clear and may not be practical: how might a 'losing' sender cell would be defined from a 'non-losing' sender cell? How could one separate the receivers from either? If receiver and sender cells come in contact during the generation of embryo chimeras, could that add noise to the system? Perhaps cell lineage specific drivers, coupled with Dox control, may be more appropriate?

Just to give one example: one could engineer a 'loser' mutation (BMPR, cMyc etc) into sender cells and then use SynNotch to profile the changes in their healthy neighbours with the aim of revealing the molecules that healthy neighbours use to 'kill' loser cells. We agree that lineage-specific drivers could be very useful for controlling timing of labelling in vivo for experiments of this type.

This is just one of many possible examples (most of which we won't have thought of!) As we note in our previous point, we are concerned that our manuscript has already exceeded the word limit by >1000 words (and we are also reluctant to constrain people's ideas by discussing specific examples of experiments, such as the one above), so we prefer not to include this information in

© 2022. Published by The Company of Biologists under the terms of the Creative Commons Attribution License (https://creativecommons.org/licenses/by/4.0/).

our discussion, if this is acceptable to the reviewer and editor.

[3.6].The authors should comment on the availability, deposition in repositories and distribution of the generated lines.

Plasmids have been deposited on Addgene and will be made available upon publication. Cell lines will be available on request. This is noted in the materials and methods section of the revised manuscript in the "DNA constructs" and "Cell Lines" sections.

Minor/editorial comments:

[3.7]
1. Figure 1: Present an overview of Notch signaling to place this tool into context. At present this is hard to understand for the non specialists.

Thank you for this suggestion. We have added this to Fig. 1 (new Fig. 1A)

[3.8]
2. Figure 1: label the nucleus vs. cytoplasmic compartment at least in one of the cell diagrams.

Good idea. Done.

[3.9]
3. Line 101 – 104, it is still possible to get multi-copy integration with electroporation. Other methods (southern blot or sequencing) would be needed to ensure the integration of a single copy.

We agree. We have removed all claims about single copy integration

[3.10]
4. Line 116. Unclear what the purpose of the HA/Myc tag on the EGFP is? A brief description is provided later, but does not clarify what can be achieved with these tags that cannot be achieved with EGFP (e.g. sorting using direct fluorescence or indirectly using antibodies).

The HA or Myc tags serve as optional additional markers (in addition to GFP) to identify sender cells, but are not essential. We have added this point to the methods section (see highlighted text in the "Cell Lines" section.

[3.11]
5. Please change eGFP to EGFP (current correct nomenclature?).

Done. Please note that we did not highlight changes to the text for these particular changes.

[3.12]
6. Line 128. Did the authors also stain for HA?

Yes. We now provide these data in new panel J in Fig. 2

[3.13]
7. Line 245-265: Quantification of induction dynamics. The authors explore kinetics using time-lapse imaging, and overall population changes using FACS.
These two approaches are complementary, but perhaps the imaging is more accurate because it is inherently at single-cell resolution which is important in this
context. However, the authors do not provide quantifications, statistics, or detailed methodology of how they reached their imaging-based conclusions. Please
provide how many cells were tracked, the variance in induction dynamics, the tracking method (manual, semi-automated or fully automated).

© 2022. Published by The Company of Biologists under the terms of the Creative Commons Attribution License (https://creativecommons.org/licenses/by/4.0/).

We have now performed a much more detailed time-lapse analysis including measuring expression of mCherry BFP and GFP within individual cells tracked in live cultures over 24h. This new data has generated new panels in Fig. 4G and in Fig. S14F-H that provide high resolution information on induction dynamics. Information about the number of cells tracked in each experiment is provided in the Figure legend and the tracking method is described in the methods ("Live imaging" section)

[3.14]
8. Line 275-279: Given the authors' data, two factors can affect the reporter (mCherry) signal intensity: (1) duration of the contact, (2) time lapsed since the contact. Thus, a weak signal could mean one of three possibilities: (1) a long interaction that ended many hours ago, (2) a short recent interaction that just ended, or (3) a newly established interaction. Presumably it would not be possible for the system to discriminate between these possibilities. Please clarify how this might be possible or include this as a caveat of the system.

This is correct. We have added this point to the discussion as follows: "We note that mCherry signal intensity will be influenced not only by the duration of contact but also where cells have moved apart, by the time lapsed since last contact: this may complicate interpretation of data from this system for some applications."

[3.15]
9. Line 323: Harnessing the system for cell fate induction. These results are exciting. Given the increasing body of literature demonstrating that mechanics impact signaling and can drive mESC fate decisions, it may be important to show a control for this experiment using a normal non-membrane GFP to prove that the mechanical strain at the border of these two populations is not driving the observed fate change.

We have now performed this control experiment and confirm that there is no neuronal differentiation when inducible-Neurog1 receivers are cultured with "fake- sender" cells. This new data is shown in Fig. S21A.

We thank both reviewers for their constructive comments and helpful suggestions.

<u>Second decision letter</u>

MS ID#: DEVELOP/2021/200226

MS TITLE: SyNPL: Synthetic Notch pluripotent cell lines to monitor and manipulate cell interactions in vitro and in vivo.

AUTHORS: Mattias Malaguti, Rosa Portero Migueles, Jennifer Annoh Annoh, Daina Sadurska, Guillaume Blin, and Sally Lowell

I have now received all the referees reports on the above manuscript, and have reached a decision. The referees' comments are appended below, or you can access them online: please go to BenchPress and click on the 'Manuscripts with Decisions' queue in the Author Area.

The overall evaluation is positive and we would like to publish a revised manuscript in Development, provided that the referees' comments can be satisfactorily addressed. You will see that Referee 2 has some helpful suggestions for clarifications. I also agree with this reviewer that including data address epigenetic silencing of the constructs would be helpful for readers.

Please attend to the reviewers' comments in your revised manuscript and detail them in your point-by-point response. If you do not agree with any of their criticisms or suggestions explain clearly why this is so. If it would be helpful, you are welcome to contact us to discuss your revision in greater detail. Please send us a point-by-point response indicating your plans for addressing the referee€™s comments, and we will look over this and provide further guidance.

© 2022. Published by The Company of Biologists under the terms of the Creative Commons Attribution License (https://creativecommons.org/licenses/by/4.0/).

Reviewer 2

Advance summary and potential significance to field

In this manuscript submitted as a resource/technical article, Malaguti and colleagues generate stable mES cell lines that are genetically engineered with synthetic signaling pathways of the synNotch family, both for sending synNotch signals and for receiving them. SynNotch pathways are used to engineer synthetic cell-cell communication pathways between ligand-producing sender cells, and receptor-expressing receiver cells. To achieve this, the authors introduce genes for anti-GFP-synNotch receptors and for synNotch-inducible target genes (mCherry and Neurog1) alongside reporter genes to generate receiver cells; and genes for membrane-GFP to generate sender cells.
One major advance of this work is the demonstration of a very powerful way to introduce synNotch pathways in stem cells, which has not been reported before. SynNotch pathways have been used in a variety of cellular and in vivo contexts, but some reports indicated challenges with signal-to-noise ratio, prompting the development of new-generation receptors. The authors show here that some of the signal-to-noise issue could be addressed by carefully selecting promoters and genetic integration approaches to generate cell lines that respond with quasi-100% activation to stimulation.
These cell lines could be used in the field both to report on neighboring relationship for understanding neighboring relationship in developmental systems (organoids or embryos), and also, for forcing differentiation to control patterning and morphogenesis for enhancing control of organoids production.

Another advance in the paper is the quality of reporting both positive and negative "dead ends", and extensive primary data for a number of different clones, which, if it became the state of the art in the field, would substantially increase reproducibility and speed up progress.

The advancement of this paper lies in the authors' demonstration of a way to generate cell lines with highly controlled activation of synNotch pathways. These cell lines could have a widespread use in the community and could allow the reach of this novel synthetic biology tool outside the domain of strictly synthetic biology laboratories more into the developmental biology research.

Comments for the author

I am very impressed with the speed and quality of the revision; the authors have addressed all the major comments I had, and majority of the minor ones. I do think the paper is ready to move forward towards publication in my opinion.
Only minor things I noticed: I do not personally think that having too many supplementary figures is a problem these days, if they can add value to the communication (not sure what the editors' opinion on this is though...). On my end, I would encourage the authors to consider including the data on the other clones that they mention they have already collected for example.
Another mention along these lines is for Fig. R1: epigenetic silencing and stability of synthetic genetic circuits in mammalian cells in general, and in stem cells in particular, is a particularly important area of focus for the community these days: in the earlier days it has been plagued with rumors and un-substantiated word-of-mouth optimization; any and all the data that can help move the field out of that phase and into a phase where the discussions are based on data (like the beautiful one you allowed me to see in Fig. R1), would be extremely important to show.
Finally, re-reading the abstract at the end, it was a bit unclear from there if a reader on first read would expect to see a "new tool" in the sense of an optimized receptor, not in the sense of an optimized cell line/procedure for building new ones. Maybe the authors can consider increasing the clarity around that point for someone that reads the abstract and is not then disappointed to not see a new receptor described.

Reviewer 3

Advance summary and potential significance to field

The authors have satisfactorily revised their manuscript addressing all previous comments/suggestions.

© 2022. Published by The Company of Biologists under the terms of the Creative Commons Attribution License (https://creativecommons.org/licenses/by/4.0/).

Comments for the author

The authors may want to go through their final text to correct what seem to be rather a lot of typos and grammatical inconsistencies.

Second revision

Author response to reviewers' comments

Reviewer 2 Comments for the Author:
I am very impressed with the speed and quality of the revision; the authors have addressed all the major comments I had, and majority of the minor ones. I do think the paper is ready to move forward towards publication in my opinion.

Only minor things I noticed: I do not personally think that having too many supplementary figures is a problem these days, if they can add value to the communication (not sure what the editors' opinion on this is though...). On my end, I would encourage the authors to consider including the data on the other clones that they mention they have already collected for example.

> *We have added data from a third clone in the 'cherry stripe experiment" as requested in the previous round of review (revised Fig S19). We agree that this is a useful addition. All other major results already show data from three clones.*

Another mention along these lines is for Fig. R1: epigenetic silencing and stability of synthetic genetic circuits in mammalian cells in general, and in stem cells in particular, is a particularly important area of focus for the community these days: in the earlier days it has been plagued with rumors and un-substantiated word-of-mouth optimization; any and all the data that can help move the field out of that phase and into a phase where the discussions are based on data (like the beautiful one you allowed me to see in Fig. R1), would be extremely important to show.

> *We agree. We have now added in the data on epigenetic silencing that was previously shown only in our previous response to the reviewer. The new data is in Fig S18D and is discussed in the corresponding results section (line 318-321.*

Finally, re-reading the abstract at the end, it was a bit unclear from there if a reader on first read would expect to see a "new tool" in the sense of an optimized receptor, not in the sense of an optimized cell line/procedure for building new ones. Maybe the authors can consider increasing the clarity around that point for someone that reads the abstract and is not then disappointed to not see a new receptor described.

> *We agree with this. We have modified the abstract to read "a new adaptation of SynNotch technology" instead of "a new tool".*

Reviewer 3 Comments for the Author:
The authors may want to go through their final text to correct
what seem to be rather a lot of typos and grammatical inconsistencies.

> *Thank you. We have looked carefully through the text and corrected some typos and grammatical inconsistencies (these are not highlighted in the revised text).*

We thank both reviewers for the many helpful suggestions that have improved our manuscript.

© 2022. Published by The Company of Biologists under the terms of the Creative Commons Attribution License (https://creativecommons.org/licenses/by/4.0/).

Third decision letter

MS ID#: DEVELOP/2021/200226

MS TITLE: SyNPL: Synthetic Notch pluripotent cell lines to monitor and manipulate cell interactions in vitro and in vivo.

AUTHORS: Mattias Malaguti, Rosa Portero Migueles, Jennifer Annoh Annoh, Daina Sadurska, Guillaume Blin, and Sally Lowell
ARTICLE TYPE: Techniques and Resources Article

I am happy to tell you that your manuscript has been accepted for publication in Development, pending our standard ethics checks.

© 2022. Published by The Company of Biologists under the terms of the Creative Commons Attribution License (https://creativecommons.org/licenses/by/4.0/).
